# Supplementary material for: Dendritic calcium signals in rhesus macaque motor cortex drive an optical brain-computer interface
Source: Nat Commun. 2021 Jun 17;12:3689. doi: 10.1038/s41467-021-23884-5 (PMC8211867; doi:10.1038/s41467-021-23884-5)
Supplement: Supplementary file 1 — Supplementary Information [file 41467_2021_23884_MOESM1_ESM.pdf]

Supplementary Information

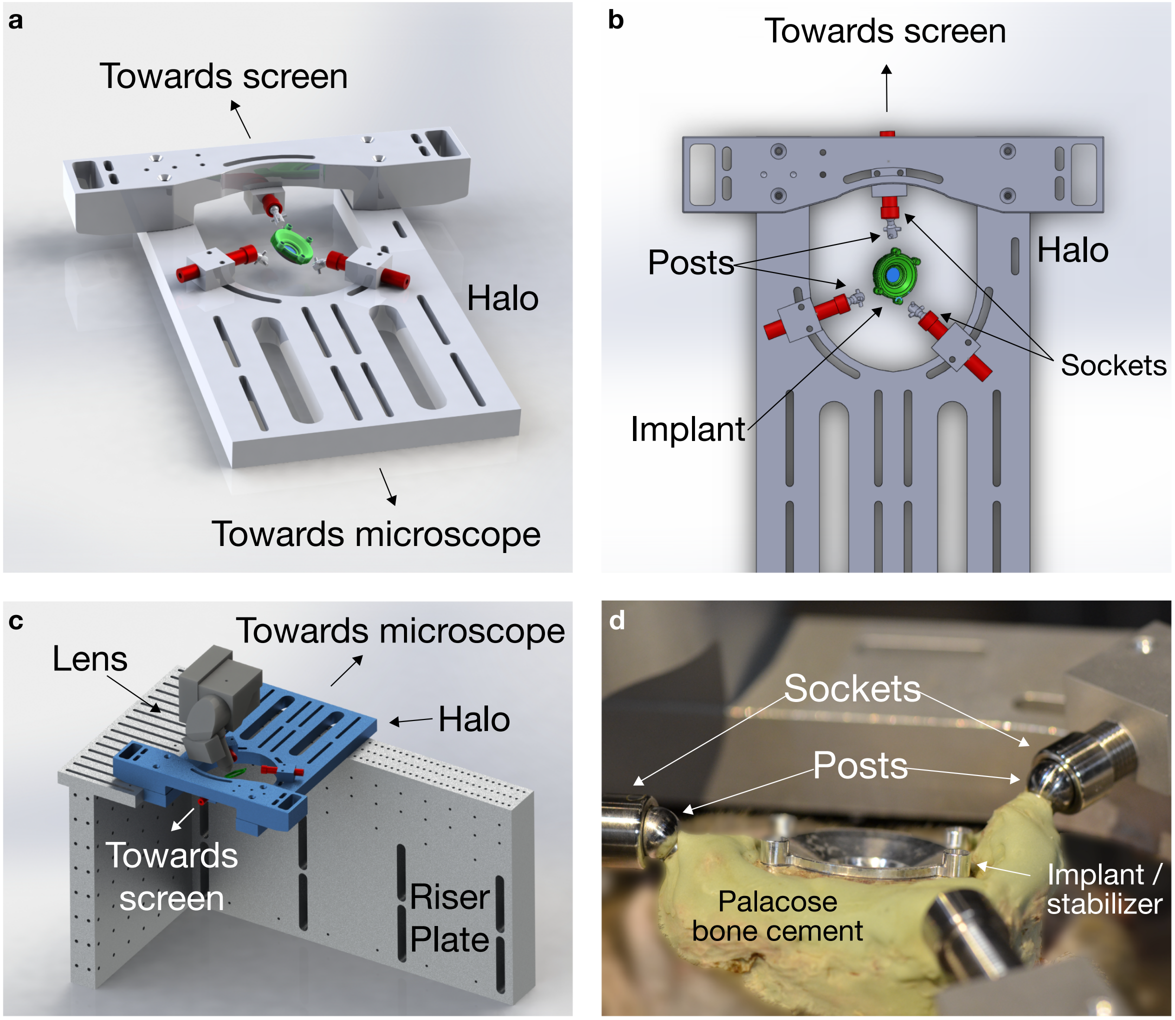

Supplementary Fig. 1: Halo and imaging stabilization. **(a, b)** Stable imaging during motor behaviors requires stabilizing the implant with respect to the optical table. We developed a custom three-point head fixation system which uses sockets to engage three stainless steel balls on posts that are embedded in bone cement in the implant. The three sockets (red) are threaded and are driven towards the implant to secure the implant in place using a hex socket on the back end of each post (threading not displayed). **(c)** The halo is fixed to the optical table via a rigid metal riser plate, consisting of a 3" aluminum plate. A 2" aluminum plate provides additional support to one side of the halo. The entire pictured assembly mounts to the front of a custom 4'x6' optical table with a ½" steel plate with mounting holes provided on one side to attach the riser plate and other hardware. (**d)** Implant for monkey X mounted in the Halo with the tissue stabilizer placed in the implant in the imaging configuration.

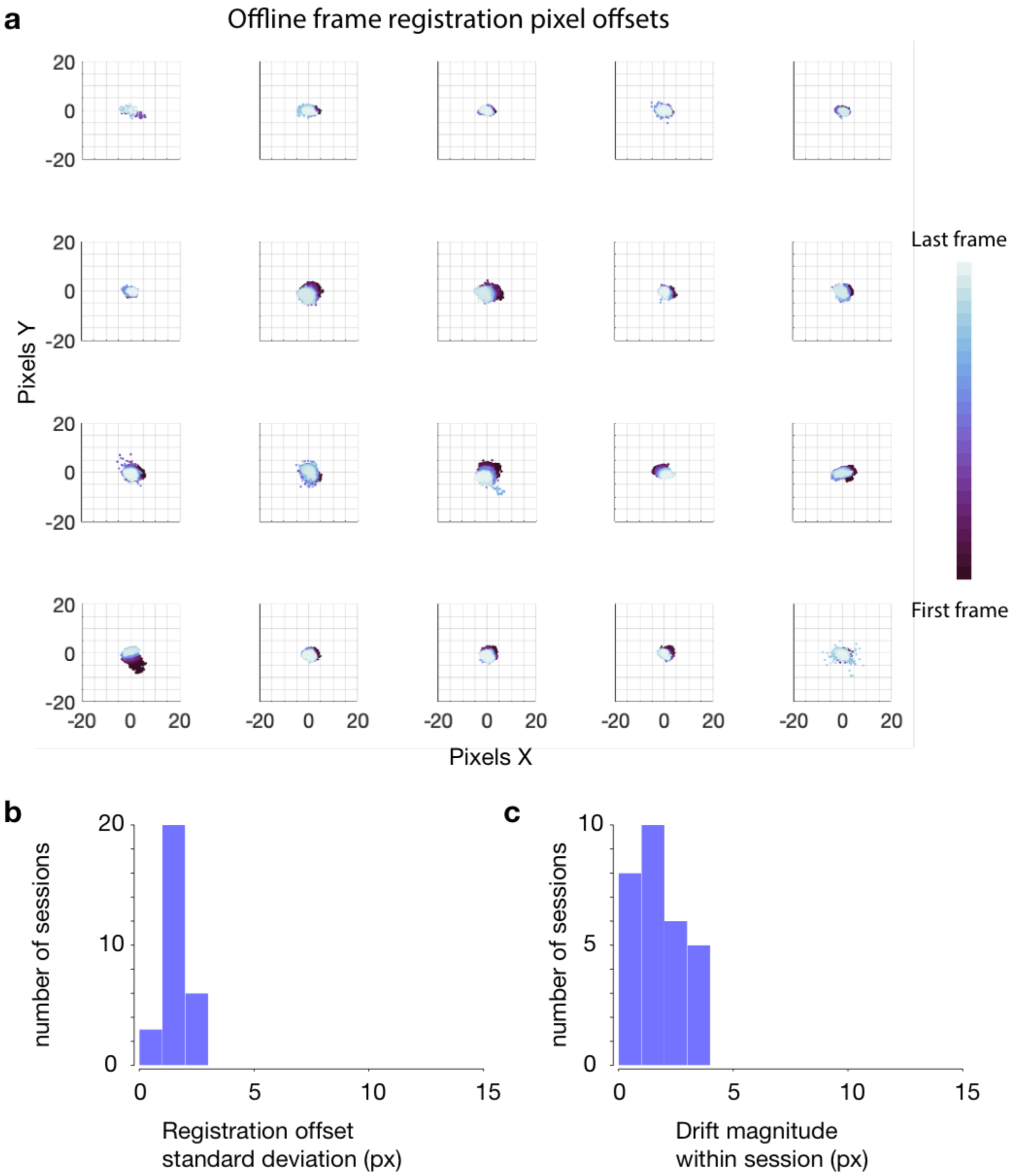

Supplementary Fig. 2: Image stability during motor behavior. (**a)** X and Y pixel offsets after image registration. Most frames require only one or two pixels worth of shift, illustrating the high degree of image stability during behavior. (**b)** Histogram of the standard deviation of the 2D distribution of X/Y pixel offsets for all datasets. **(c)** Histogram of the magnitude of X/Y drift measured between the first 25% of frames and last 25% of frames within single image series.

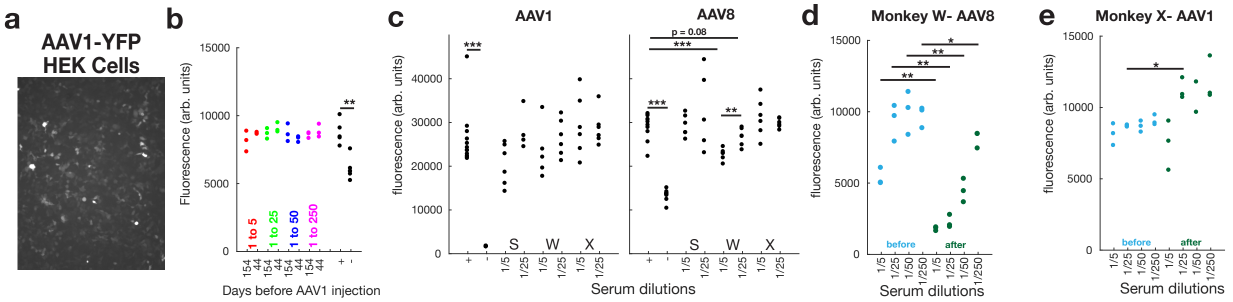

Supplementary Fig. 3: Neutralizing antibody assay **(a)** Example image of AAV1-Ef1a-YFP infected HEK cells. **(b)** Neutralizing antibodies were stable across 100 days and multiple serum dilutions (ANOVA, $F_{7,16}$ = .96, p = 0.49) (n=3 replicates per experimental condition). Positive and negative controls (no serum / no virus) were significantly different (p = 4.7444e-04, unpaired two-sided t-test) **(c)** Each macaque subject (S, W and X) had pre-existing neutralizing antibodies to different AAV serotypes. AAV1 control: p=2.86e-11 (one-sided t-test), AAV8 control: 1.06e-13 (one-sided t-test); Monkey W, AAV8 1/5 dilution: p=0.00021809; Monkey W, AAV8 1/5 vs 1/25 dilutions: p=0.00949298 (two-sided t-test). **(d)** Monkey W developed significant antibodies to AAV8 post-injection (unpaired two-sided t-test, dilution 1/5: p=0.0017; 1/25: p=0.0017; 1/50: p=0.0068; 1/250: p=0.043, not adjusted for multiple comparisons). **(e)** Monkey X did not develop significant antibodies to AAV1 post-injection. (unpaired two-sided t-test, dilution 1/5: p=0.3; 1/25: p=0.01; 1/50: p=0.11; 1/250: p=0.06, not adjusted for multiple comparisons). All panels: * p < 0.05, ** p < 0.01, *** p < 0.001. Source data and statistical results are provided as a supplemental source data file.

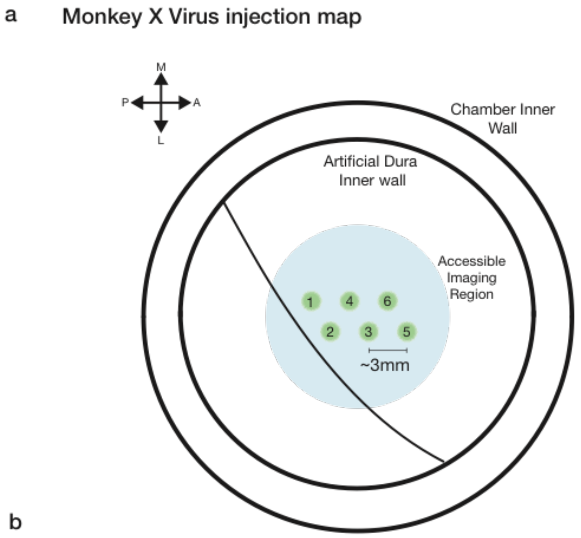

b

1. AAV1-CaMKIIα-GCaMP6f, 3.9e+13, 750nL @1mm and 750nL @1.5mm
2. AAV1-CaMKIIα-GCaMP6f, 3.9e+13, 400nL @0.8mm and 400nL @1.2mm
3. AAV1-CaMKIIα-mGCaMP6f-NES, 4.7e+13, 750nL @0.8mm and 750nL @1.3mm
4. AAV1-CaMKIIα-mGCaMP6f-NES, 4.7e+13, 400nL @0.8mm and 240nL @1.3mm
5. AAV1-hSyn-GCaMP5g-NES, 6.3e+12, 750nL @0.8mm and 286nL @1.3mm
6. AAV1-hSyn-GCaMP5g-NES, 6.3e+12, 400nL @0.8mm and 400nL @1.3mm

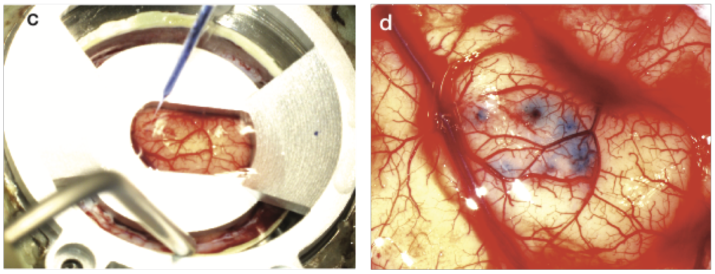

Supplementary Fig. 4: Virus injection map and cortical surface pictures for Monkey X. **(a)** Viral construct injection map. **(b)** List of injected viral constructs and injection volumes and concentrations **(c)** Tissue stabilization apparatus and glass pipette during injections (**d**) Injection sites from (a), visible via Trypan blue dye mixed with viral constructs.

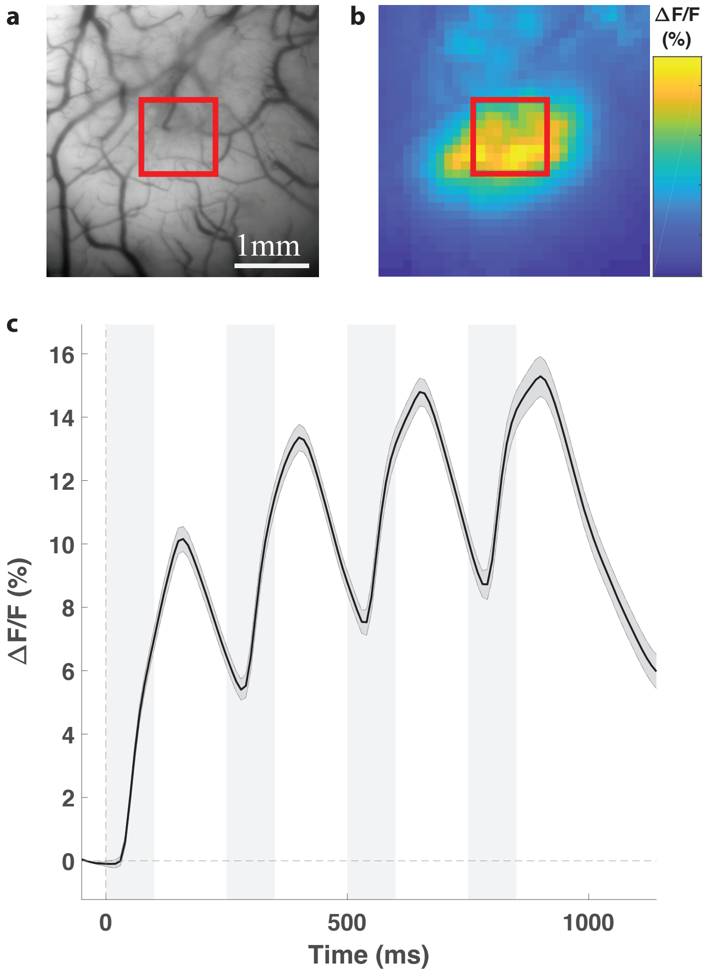

Supplementary Fig. 5: Reliable widefield GCaMP signal from macaque visual cortex (Monkey L). **(a)** Reference vasculature of imaged area. **(b)** widefield signal in response to 4Hz flashed grating (right) at the intersection of two sites injected with AAV1-CaMKIIα-NES-GCaMP6f. In the response map, color indicates amplitude of the 4 Hz FFT component computed at each location. **(c)** Average time course of GCaMP response to a 4 Hz flashed grating (100 ms on, 150 ms off) with stimulus presentation time shaded in gray. Shaded area around the time course signal represents ± SEM over 10 stimulus repetitions. Note that the GCaMP signal does not quickly return to baseline after each stimulus presentation, so a buildup in the response can be seen over time, as reported previously with CaMKIIα-GCaMP[21]. The 1x1 mm ROI used for the time course is indicated by red squares in (a). Example signal was recorded 14 weeks post-injection and continues to provide reliable signal after 13 months.

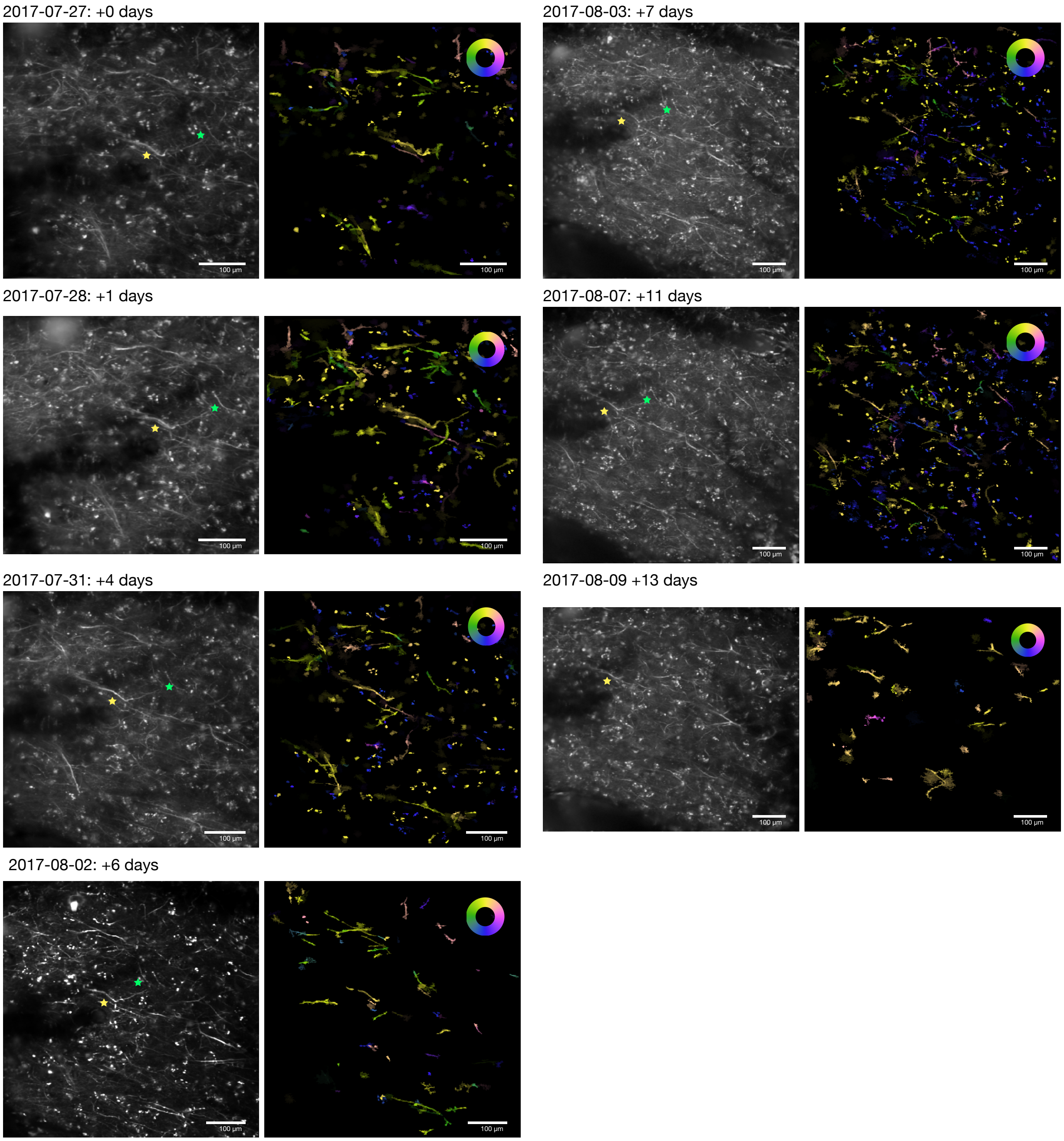

Supplementary Fig. 6: Neural features identifiable across experimental sessions. Each pair of images corresponds to an individual imaging session, all of which were collected at highly overlapping fields of view. Left panels show enhanced mean images; right panels show ROI tuning maps. Individual dendritic / axonal processes can be identified using a combination of their idiosyncratic morphology and their location relative to the consistent shadow created by superficial vasculature. Two examples are identified with yellow and green stars in each image where they are visible.

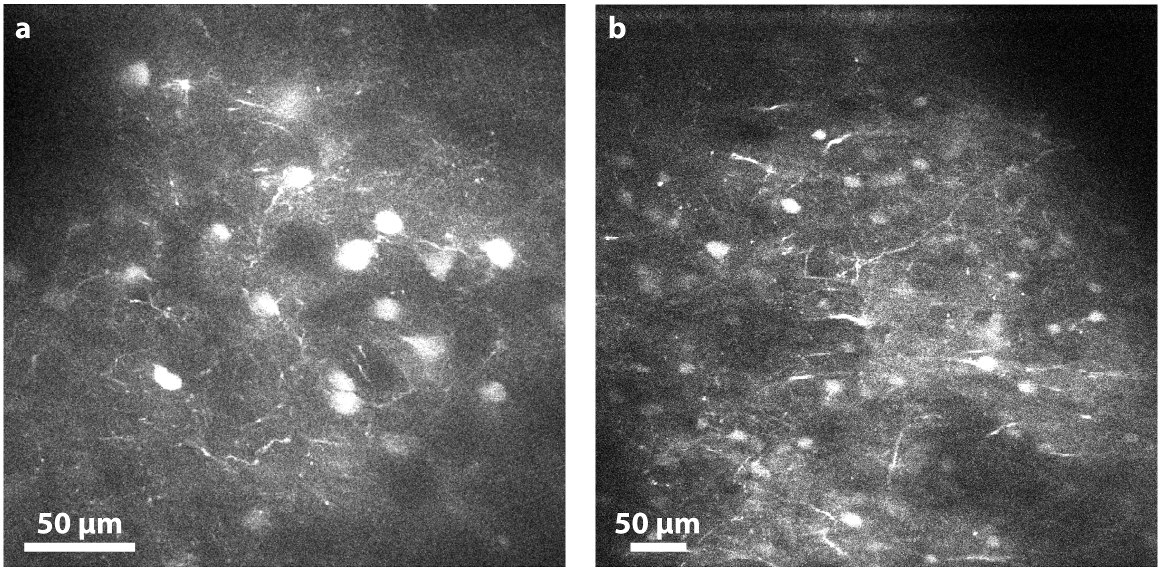

Supplementary Fig. 7: Cells exhibiting non-standard GCaMP expression. **(a,b)** Two fields of view from Monkey W showing numerous brightly fluorescent cells that do not exhibit a halo pattern characteristic of typical calcium imaging. These cells do not modulate in fluorescence during behavior. We observed this phenomenon at multiple injection sites and multiple subjects and a range of viral serotypes, including AAV1 and AAV with CAMKIIa and hSyn promoters. While we would suspect unhealthy overexpression of GCaMP due to filled nuclei and lack of traditional halo shape, we observed the presence of these cells within six weeks of injecting virus. In addition, such cells did not exhibit healthy levels of fluorescence before displaying bright, filled nuclei. Instead, they exhibit no fluorescence and appeared as shown within several days. This suggests that overexpression may not be the primary explanation for this phenomenon. These two images are representative of the expression patterns observed in approximately ~20 imaging sessions in Monkey W.

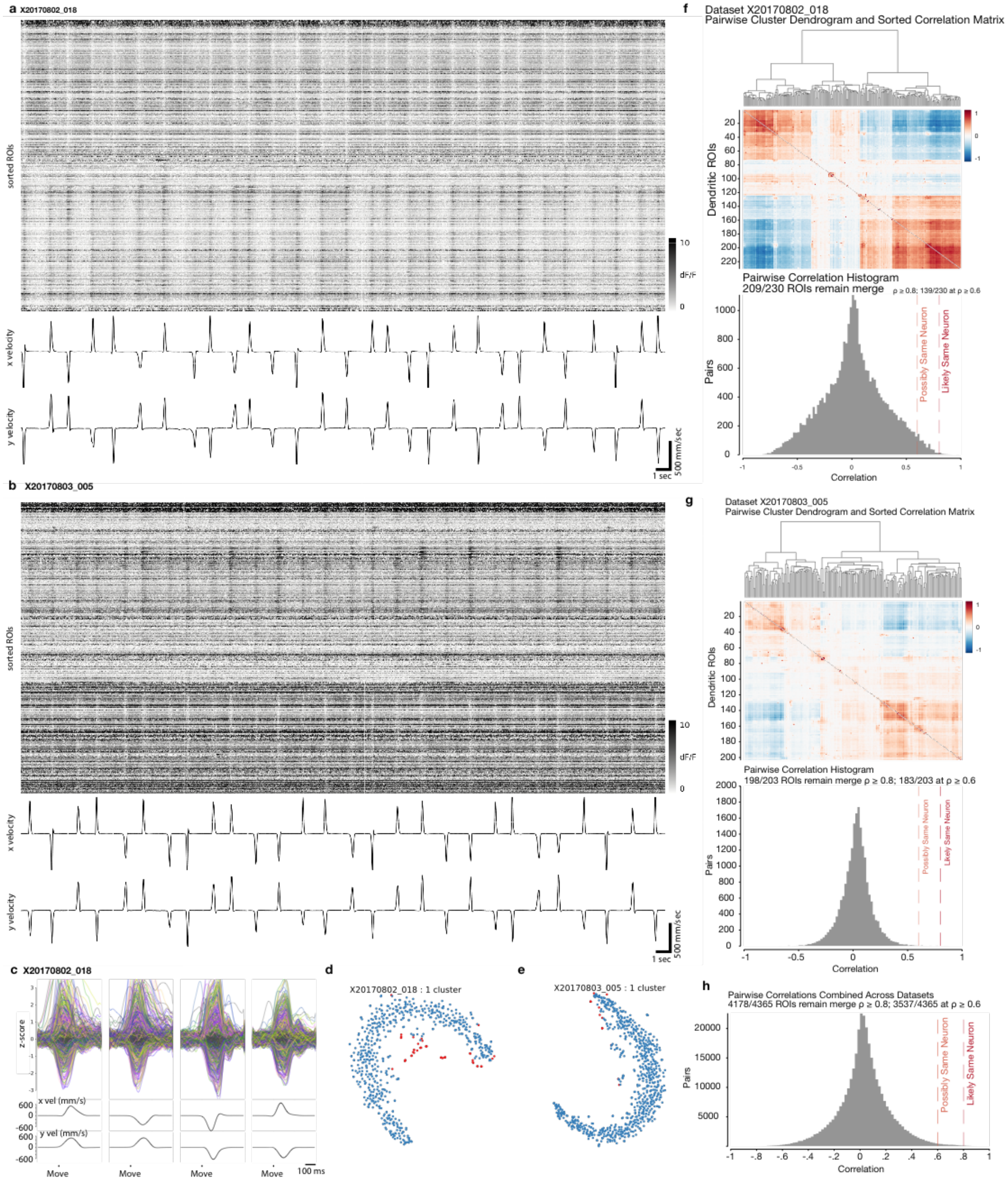

Supplementary Fig. 8: Single trial raster plots of all ROIs showing significant modulation during movement (see Methods). **(a)** Each row represents the activity of a single ROI over time as ΔF/F relative to baseline. The rows of the raster are sorted vertically using rastermap (https://github.com/MouseLand/rastermap), a multi-dimensional embedding algorithm that groups ROIs

with highly similar responses close together to reveal shared structure across cells. This shared structure across ROIs is expected given the low-dimensionality of motor cortical neural responses observed using extracellular electrophysiology. Below the raster are the hand velocity in X and Y, including reaches from the center to the target and the reach back to the center position. Short breaks in the hand trace represent isolated missing hand position samples; time runs continuously in this plot. **(b)** Same as (a) but for a second imaging session at a different field of view. **(c)** Diversity of ROI responses during movement. Top row shows trial-averaged ROI responses aligned to movement onset. Colored traces represent ROIs with a statistically significant peri-movement response; dark gray traces indicate ROIs with non-significant peri-movement responses. Each column shows the same ROIs for each of the four reaching directions, with the trial-averaged X,Y hand velocity components plotted beneath. **(d,e)** Results of an analysis demonstrating that individual ROI responses form a continuum rather than discrete clusters. (see Methods). Each point represents an individual ROI, plotted in a two-dimensional embedding identified by t-SNE. Blue points belong to the single cluster identified automatically using the DBScan clustering algorithm; red points were identified as noise outside of any clusters. The same result was found for all 36 datasets. **(f, g)** Results of an ROI pairwise correlation analysis described in Peters et al. (2017) to identify ROI pairs that exhibit sufficiently correlated dendritic responses to be considered as originating from the same neuron. Top row: Dendrograms were computed using hierarchical clustering, and the order of ROIs was optimized to maximize the sum of the similarities between adjacent ROIs. Distances between each pair of ROIs were computed as d = (1 - correlation). Middle: correlation matrix between ROI signals, using the optimized ROI ordering. Bottom row: histogram of pairwise correlation coefficients between pairs of ROIs against the threshold ρ=0.8 used in Peters et al. (2017) and a more conservative threshold of ρ=0.6. **(h)** Same histogram as (g) but across multiple datasets.

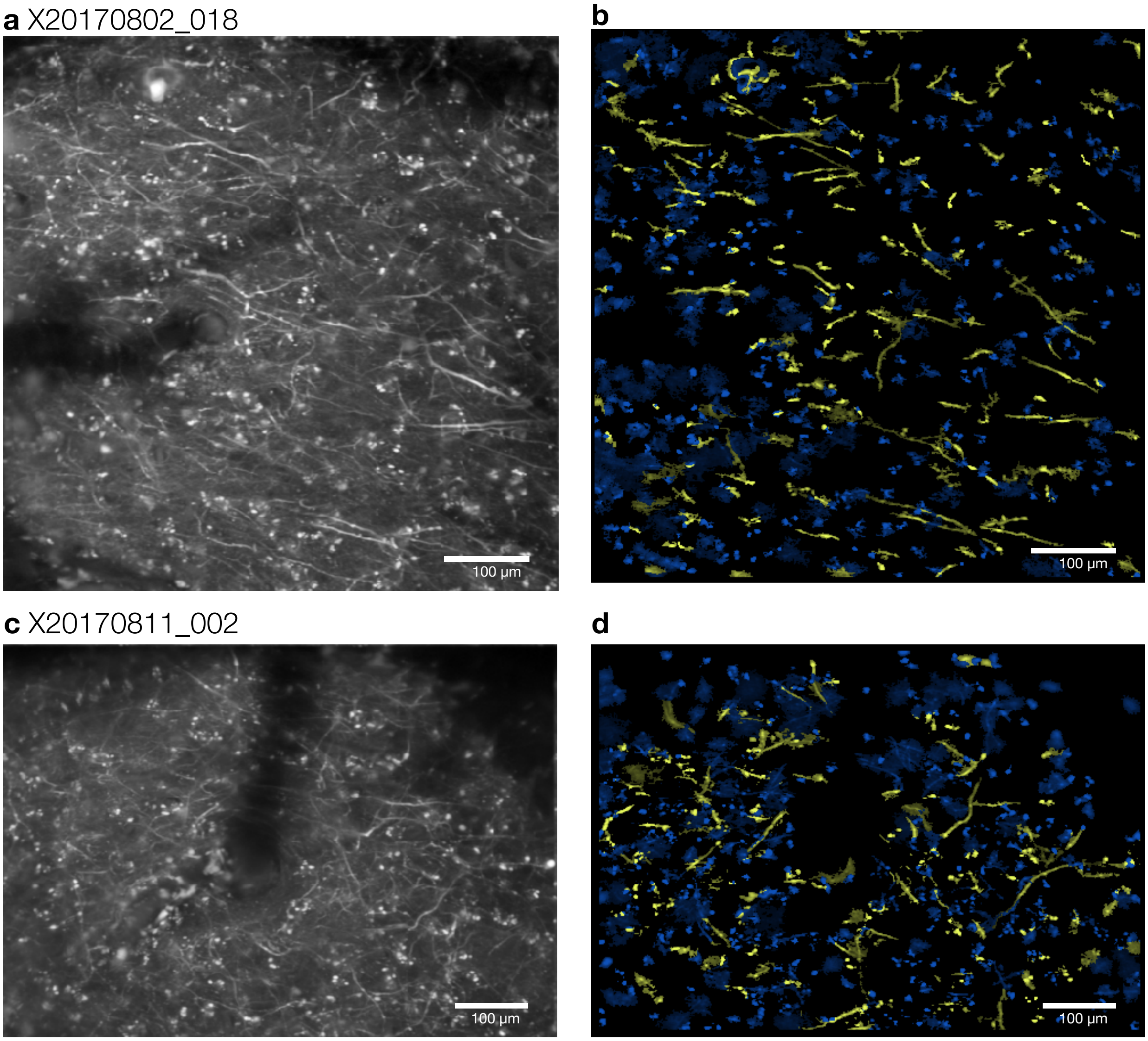

Supplementary Fig. 9: Demonstration of identified putative dendritic/axonal ROIs: **(a, c)** Enhanced mean images from two example imaging sessions. **(b, d)** Yellow pixels show identified dendritic/axonal ROIs; blue pixels show ROIs not identified as dendritic/axonal. Representative images from one of 36 analyzed sessions.

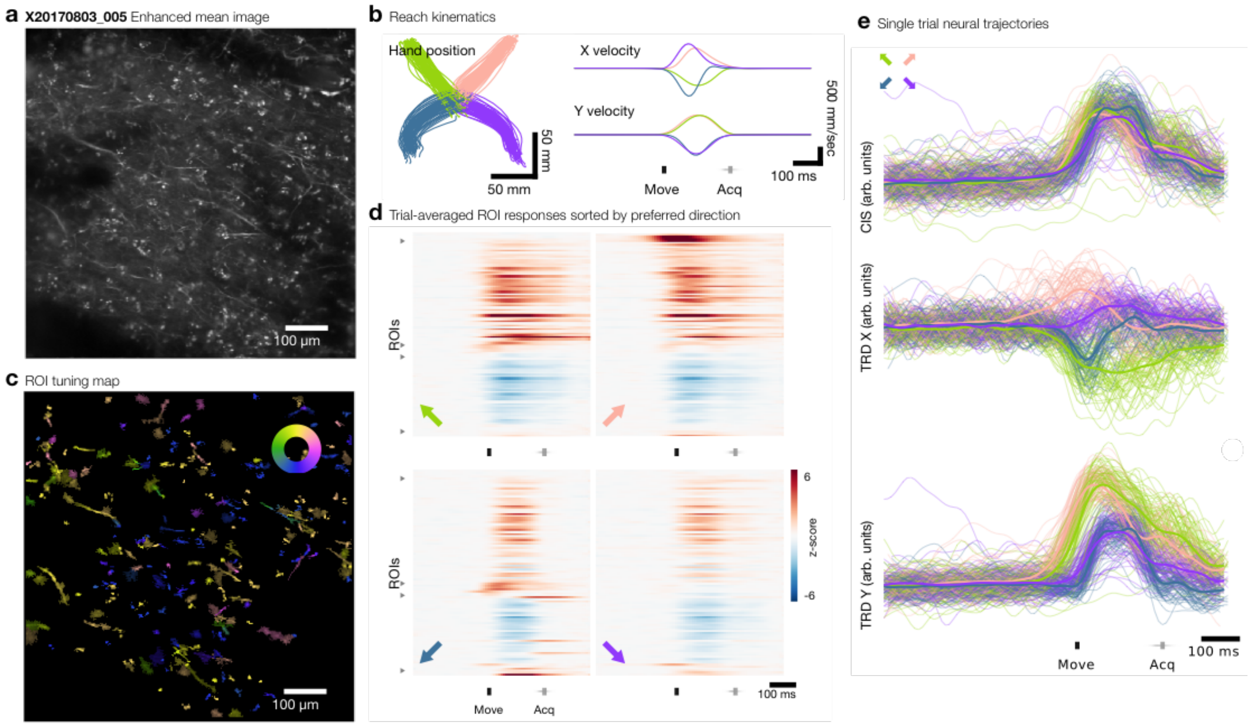

Supplementary Fig. 10: Functional responses during reaching behavior. Same format as Fig. 5 in main manuscript but for a different field of view.

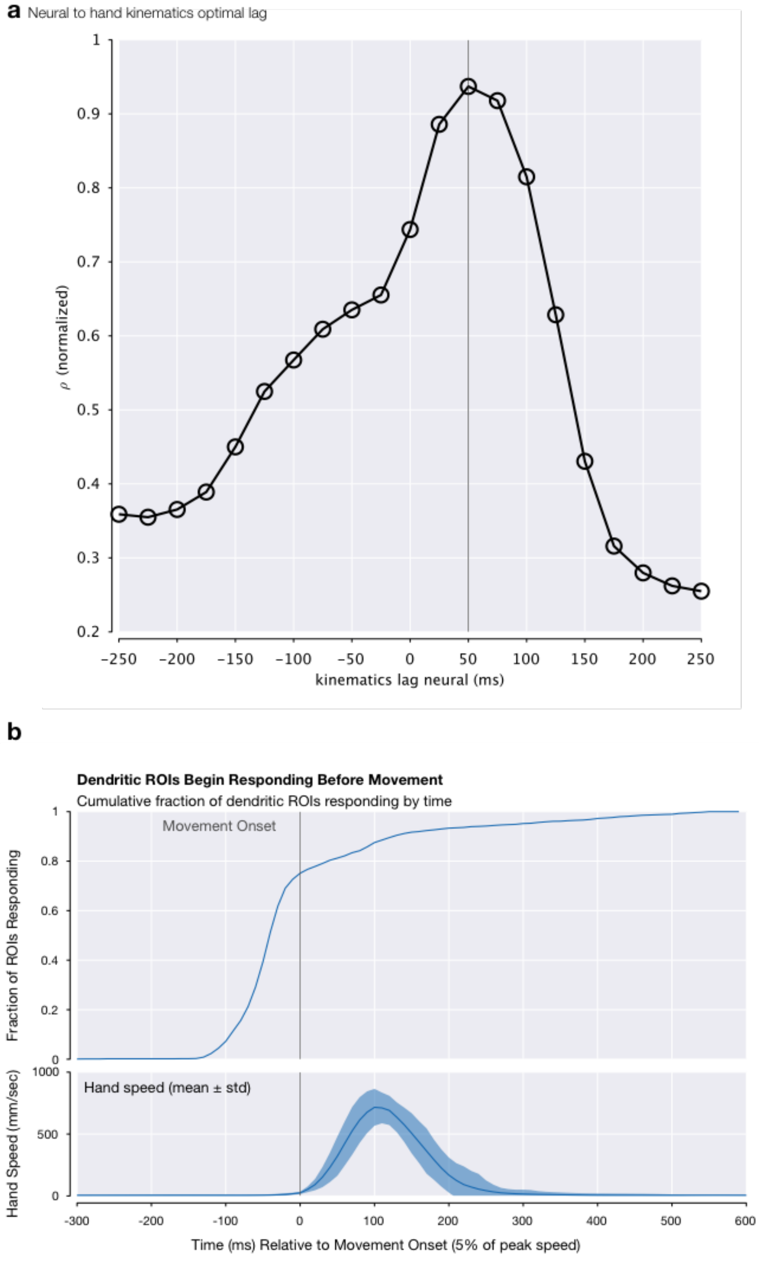

Supplementary Fig. 11: **(a)** Optimal neural to kinematics regression lag. For each imaging session the median correlation coefficient across each ROI and X and Y velocity was computed as a function of lag. Each curve was then normalized to a maximum of 1 and then averaged across sessions. The empirical maximum at 50 ms implies that neural responses lead kinematic variables by 50 ms. **(b)** Top panel: cumulative proportion of the 2196 tuned dendritic ROIs that exhibit directionally tuned responses at or before a given time point relative to behaviorally defined movement onset (t=0). The time at which a given ROI exhibits a directionally tuned response is determined by performing a Kruskal Wallis nonparametric one-way analysis of variance on the single-trial fluorescence measurements at each time in a sliding window. The response time is taken when significance at $p < 0.01$ is reached for 5 consecutive 10 ms windows. Bottom panel: hand speed (mean ± std.) aligned to behaviorally defined movement onset for the same trials.

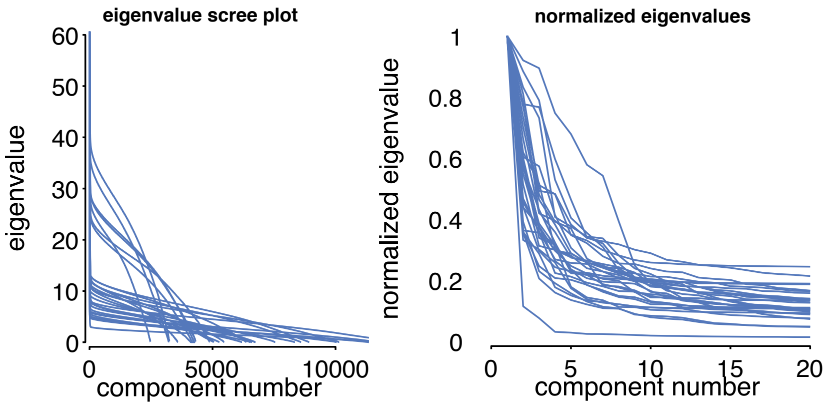

Supplementary Fig. 12: Left: Eigenvalue scree plot of dF/F imaging data, multiple sessions. **Right:** normalized eigenvalues shown with restricted x-axis range to illustrate knee in the scree plot.

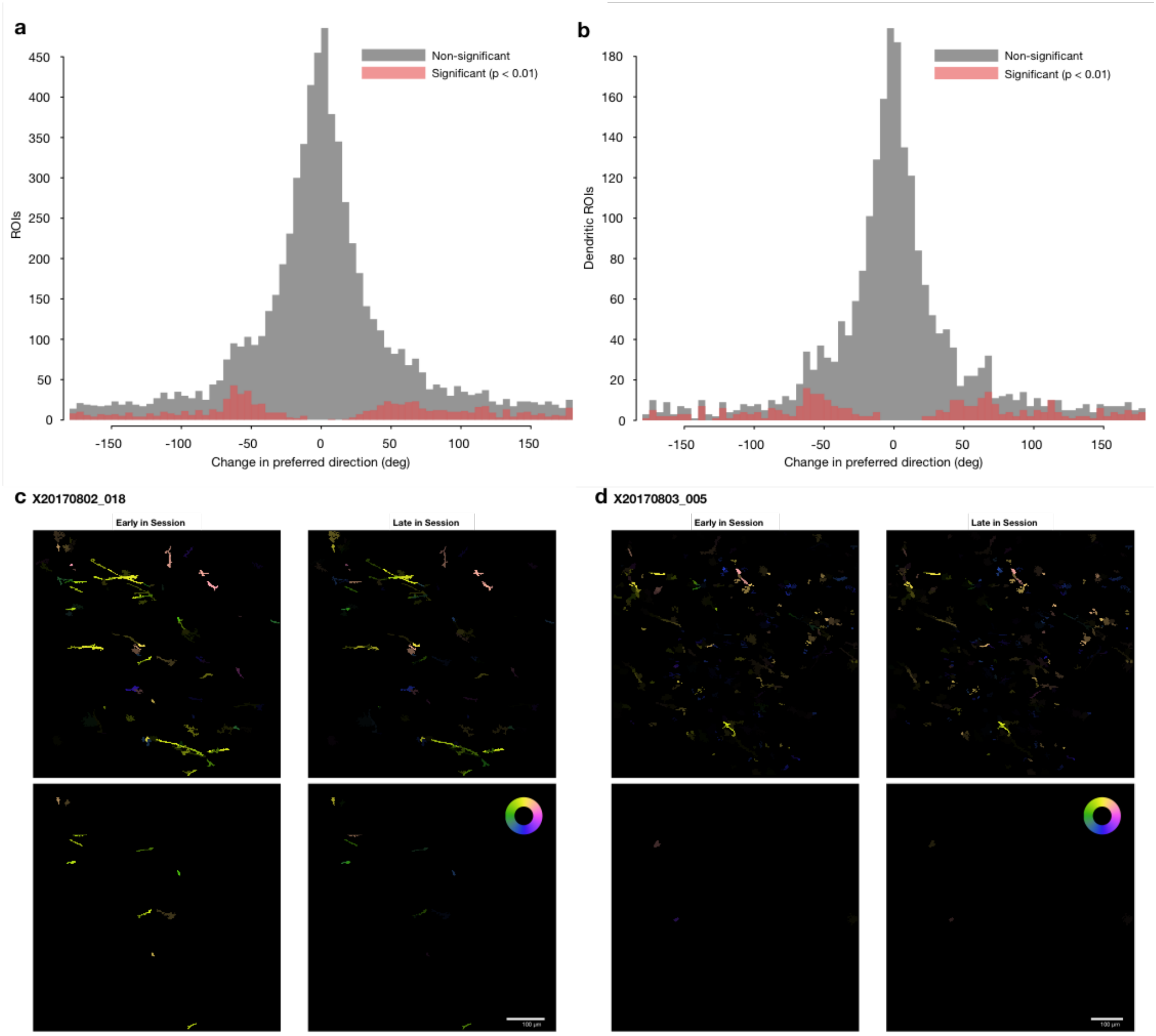

Supplementary Fig. 13: Consistent tuning within recording sessions. **(a)** Histogram of observed changes in preferred direction for all 2196 direction-tuned ROIs measured at the last 25% of each recording session relative to the first 25% of each recording session. Red histogram shows statistically significant changes (p < 0.01, two-sided permutation test, unadjusted). **(b)** Histogram of observed changes in preferred direction for all direction-tuned, putative dendritic / axonal ROIs. **(c, d)** Two example ROI tuning maps measured in the first 25% (left) and last 25% (right) of each session. All direction-tuned ROIs are shown in top panels; only those with significant changes in preferred direction are shown in the bottom panels.

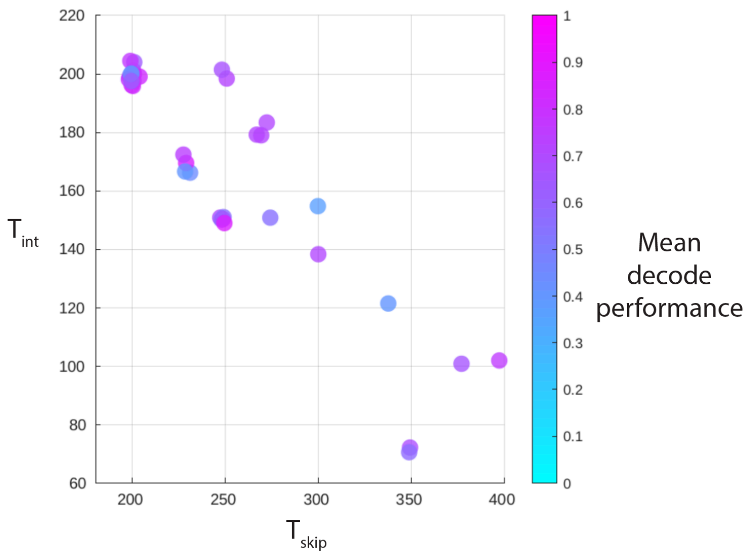

Supplementary Fig. 14: Parameter values used for online decoding sessions. Tskip is the time after go cue ignored by the decoder. Tint is the integration time over which the decoder processes streaming images before selecting the maximum likelihood target.

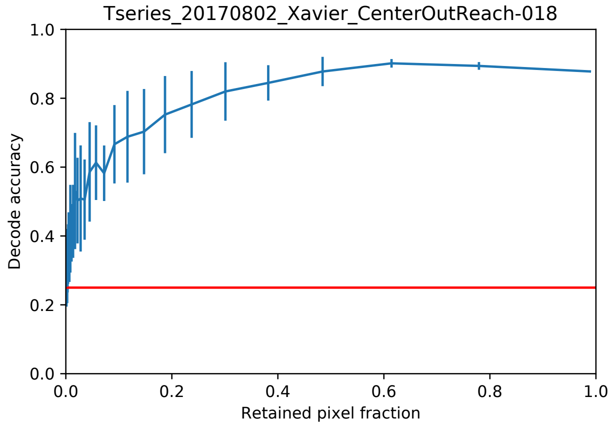

Supplementary Fig. 15: Impact of masking out pixels on offline decode performance for an example session (Mean +/- SEM, n = 5 iterations, see Methods for analysis details).

Supplementary Tables

Table 1 – Injection and serum collection details

Subject	Injection Date	Viral Serotypes	Date, Pre-injection Serum	Date, Post-injection Serum	Date, others tested	NAB Serotypes Tested
S			11/17/14	6/01/15		AA8
W	11/09-11/13/2015	AAV1, AAV8	10/20/2015	11/24/2015		AAV1, AAV8
X	4/07/2017	AAV1	11/04/2016	7/10/2017	04/07/2017	AAV1, AAV8
L	5/25-12/29/2017	AAV1, AAV5, AAV8, AAVdj	4/25/2017	11/29/2017		

Table 2 – viral titers used in immunoreactivity assay

Virus	Original Titer	Diluted titer used
AAV1-Ef1α-YFP	1.8 x 10^13	1.8 x 10^11
AAV8-Ef1α-eYFP	2.4 x 10^14	2.4 x 10^12
