# Supplementary material for: Dendritic calcium signals in rhesus macaque motor cortex drive an optical brain-computer interface
Source: Nat Commun. 2021 Jun 17;12:3689. doi: 10.1038/s41467-021-23884-5 (PMC8211867; doi:10.1038/s41467-021-23884-5)
Supplement: Supplementary file 3 — Description of Additional Supplementary Files [file 41467_2021_23884_MOESM3_ESM.docx]

**Description of Additional Supplementary Files**

File Name: Supplementary Movie 1 (example_dendrite_imaging_201708_short)

Description: Example in-vivo two-photon calcium imaging data containing dendritic processes in rhesus macaque motor cortex during a reaching task.

File Name: Supplementary Movie 2 (4-condition decode - muted.mp4)

Description: Example trials from the reaching behavioral task with simultaneous real-time decode of the monkey’s reach target from the calcium imaging data.

File Name: Supplementary Movie 3 (CLARITY imaging of tissue volume.mp4)

Description: Example post-hoc CLARITY volumetric reconstruction of GCaMP-expressing neurons and dendritic processes in rhesus macaque motor cortex.

File Name: Supplementary data 1

Description: Supplementary data file with source data accompanying manuscript figures.
