# Supplementary material for: Dendritic calcium signals in rhesus macaque motor cortex drive an optical brain-computer interface
Source: Nat Commun. 2021 Jun 17;12:3689. doi: 10.1038/s41467-021-23884-5 (PMC8211867; doi:10.1038/s41467-021-23884-5)
Supplement: Supplementary file 8 — Reporting Summary [file 41467_2021_23884_MOESM8_ESM.pdf]

natureresearch

Corresponding author(s): Eric Trautmann, Krishna Shenoy

Last updated by author(s): 9/20/2019

# Reporting Summary

Nature Research wishes to improve the reproducibility of the work that we publish. This form provides structure for consistency and transparency in reporting. For further information on Nature Research policies, see Authors & Referees and the Editorial Policy Checklist .

## Statistics

For all statistical analyses, confirm that the following items are present in the figure legend, table legend, main text, or Methods section.

| n/a | Confirmed | |
|---|---|---|
| ☐ | ☒ | The exact sample size (*n*) for each experimental group/condition, given as a discrete number and unit of measurement |
| ☐ | ☒ | A statement on whether measurements were taken from distinct samples or whether the same sample was measured repeatedly |
| ☐ | ☒ | The statistical test(s) used AND whether they are one- or two-sided<br>*Only common tests should be described solely by name; describe more complex techniques in the Methods section.* |
| ☒ | ☐ | A description of all covariates tested |
| ☒ | ☐ | A description of any assumptions or corrections, such as tests of normality and adjustment for multiple comparisons |
| ☐ | ☒ | A full description of the statistical parameters including central tendency (e.g. means) or other basic estimates (e.g. regression coefficient) AND variation (e.g. standard deviation) or associated estimates of uncertainty (e.g. confidence intervals) |
| ☐ | ☒ | For null hypothesis testing, the test statistic (e.g. *F*, *t*, *r*) with confidence intervals, effect sizes, degrees of freedom and *P* value noted<br>*Give P values as exact values whenever suitable.* |
| ☒ | ☐ | For Bayesian analysis, information on the choice of priors and Markov chain Monte Carlo settings |
| ☒ | ☐ | For hierarchical and complex designs, identification of the appropriate level for tests and full reporting of outcomes |
| ☒ | ☐ | Estimates of effect sizes (e.g. Cohen's *d*, Pearson's *r*), indicating how they were calculated |

*Our web collection on statistics for biologists contains articles on many of the points above.*

## Software and code

Policy information about availability of computer code

| | |
|---|---|
| Data collection | Imaging data was collected using PrairieView software (Prairie View 5.4 Alpha, revision 69 and later versions). Behavioral data was collected using custom MATLAB / Simulink code, including the following repositories: https://github.com/djoshea/obci, https://github.com/djoshea/matudp, ImGUI (version number not specified - https://github.com/ocornut/imgui) |
| Data analysis | Analysis was performed using custom MATLAB / Simulink and Python code, as well as the open-source NoRMCorre package(v0.1.1) (see https://www.biorxiv.org/content/10.1101/108514v2). Additional open source libraries were used Suite2P ( - https://github.com/cortex-lab/Suite2P), Sci-kit learn (v0.22.1- https://scikit-learn.org/), Rastermap (version not specified - https://github.com/MouseLand/rastermap), dPCA (no version - https://github.com/machenslab/dPCA), CET colormaps (version not specified - https://peterkovesi.com/projects/colourmaps/), libuv (version not specified - http://libuv.org/), Jupyter (version not specified - https://jupyter.org/ ), SciPy (version not specified - https://www.scipy.org/), Numba (version not specified - http://numba.pydata.org/ ), Cython (version not specified - https://cython.org/). |

For manuscripts utilizing custom algorithms or software that are central to the research but not yet described in published literature, software must be made available to editors/reviewers. We strongly encourage code deposition in a community repository (e.g. GitHub). See the Nature Research guidelines for submitting code & software for further information.

## Data

Policy information about availability of data

All manuscripts must include a data availability statement. This statement should provide the following information, where applicable:

- Accession codes, unique identifiers, or web links for publicly available datasets
- A list of figures that have associated raw data
- A description of any restrictions on data availability

The datasets generated during and/or analyzed during the current study are available from the corresponding author on reasonable request.

# Field-specific reporting

Please select the one below that is the best fit for your research. If you are not sure, read the appropriate sections before making your selection.

☒ Life sciences ☐ Behavioural & social sciences ☐ Ecological, evolutionary & environmental sciences

For a reference copy of the document with all sections, see nature.com/documents/nr-reporting-summary-flat.pdf

# Life sciences study design

All studies must disclose on these points even when the disclosure is negative.

| | |
|---|---|
| Sample size | Four primate subjects were included in this study. Three received imaging implants and viral injections in motor cortex (PMd/M1), and one primates received virus injections and imaging implants in visual cortex (V1). Neutralizing antibody assay results are reported for all four primates, and functional imaging is reported for two primates. The length of decoding experiments (number of trials) was not specified a-priori, but the significance of the decode performance was assessed in all cases post hoc. For CLARITY imaging experiments, all stained tissues were imaged. For neutralizing antibody assay, samples for all four primates were included. The sample size of four primates was selected to balance between replication, cost, and feasibility as consistent with standard practices in nonhuman primate neurophysiology. |
| Data exclusions | We did not observe functional expression of the GCaMP constructs in two primates, and did not include decoding results for those two animals. A third primate, with V1 chamber, developed health complications and we did not perform imaging with this animal, but include neutralizing antibody assay results. Online neural decoding sessions in which in which the decoder stopped functioning (due to tissue drift or other non-stationarities) were terminated at the discretion of the experimentalists after documenting performance roll-off. |
| Replication | The key technical achievement of micron-stable imaging during awake behaviors was replicated in four animals, three in PMd/M1 using two photon imaging and one in V1 using widefield functional imaging. In all animals, we demonstrated stable imaging during behavior, though functional neural signals were limited to one subject in motor cortex and one subject in V1. We replicated our decoding results using 36 decoder sessions to establish proof of concept. CLARITY imaging of ex-vivo tissue was only performed once, as we do not anticipate variability across multiple samples. The neutralizing antibody assay employed three replicates per experimental condition. All replications were successful. |
| Randomization | N/A. All comparisons were performed within subject (i.e. immunoreactivity before and after virus expression or comparison of online optical brain machine interface decoder in regions with function GCaMP expression vs. non-expression region (control). Comparisons and statistical tests were not performed across subjects, so randomization of subjects to treatment groups is not appropriate for this particular study. Randomization was not possible for ex-vivo CLARITY imaging of stained tissue samples, and is not applicable to the Neutralizing antibody assay. |
| Blinding | The operation of the online optical brain machine interface decoder was automated, requiring no intervention from the experimenter to decode neural activity and updated the stimulus and provide a reward to the animal. As such, this portion of the experiment can be considered blinded in that the experimenters had no interaction with the data collection aside from starting and stopping the decoder and recording. The experimenters are not otherwise blinded during an experimental session, but are not in the same room with the animal and are unable to influence the behavior in any way other than adjusting reward rate, which is typically kept within a narrow range during an experiment. The animal subjects are not aware of the specific manipulations or of which injection location and imaging construct is used to drive an online decoder. The ex-vivo imaging studies involved automated acquisition of the full imaging volume after staining, but were not otherwise blinded. The neutralizing antibody assay was automated but not otherwise blinded. |

# Reporting for specific materials, systems and methods

We require information from authors about some types of materials, experimental systems and methods used in many studies. Here, indicate whether each material, system or method listed is relevant to your study. If you are not sure if a list item applies to your research, read the appropriate section before selecting a response.

## Materials & experimental systems

| n/a | Involved in the study | |
|---|---|---|
| ☐ | ☒ | Antibodies |
| ☐ | ☒ | Eukaryotic cell lines |
| ☒ | ☐ | Palaeontology |
| ☐ | ☒ | Animals and other organisms |
| ☒ | ☐ | Human research participants |
| ☒ | ☐ | Clinical data |

## Methods

| n/a | Involved in the study | |
|---|---|---|
| ☒ | ☐ | ChIP-seq |
| ☒ | ☐ | Flow cytometry |
| ☒ | ☐ | MRI-based neuroimaging |

## Antibodies

| | |
|---|---|
| Antibodies used | CLARITY histology imaging performed using GFP Polyclonal Antibody, Alexa Fluor 647 (A-31852), |

| | |
|---|---|
| Validation | From the manufacturer: "The Alexa Fluor™ dyes provide for extraordinarily bright antibody conjugates. At the time of preparation, the products are certified to be free of unconjugated dyes and are tested in a cytological experiment to ensure low nonspecific staining." Certificate of analysis available online. |

## Eukaryotic cell lines

Policy information about cell lines

| | |
|---|---|
| Cell line source(s) | Human Embryonic Kidney 293, cell line internally maintained, original source is Thermofisher Scientific |
| Authentication | Cells were authenticated by the vendor |
| Mycoplasma contamination | Not tested |
| Commonly misidentified lines (See ICLAC register) | None |

## Animals and other organisms

Policy information about studies involving animals; ARRIVE guidelines recommended for reporting animal research

| | |
|---|---|
| Laboratory animals | This study included four adult male monkeys (macaca mulatta): S (California National Primate Research Center, age: 8); W (Oregon National Primate Research Center, age: 10); X (Oregon National Primate Research Center, age: 11); L (University of Louisiana at Lafayette, New Iberia Research Center, age: 6) |
| Wild animals | No wild animals were used in this study. |
| Field-collected samples | No field-collected samples were used in this study. |
| Ethics oversight | All procedures and experiments were approved by the Stanford University Institutional Animal Care and Use Committee (Monkeys S,W,and X) or the University of Texas Institutional Animal Care and Use Committee (Monkeys L) and were performed in compliance with the Guide for the Care and Use of Laboratory Animals and conform to NIH standards. |

Note that full information on the approval of the study protocol must also be provided in the manuscript.
